# Ultrasound Exposure Improves the Targeted Therapy Effects of Galactosylated Docetaxel Nanoparticles on Hepatocellular Carcinoma Xenografts

**DOI:** 10.1371/journal.pone.0058133

**Published:** 2013-03-01

**Authors:** Hongfen Wei, Jing Huang, Jing Yang, Xiujuan Zhang, Liwu Lin, Ensheng Xue, Zhikui Chen

**Affiliations:** 1 Department of Ultrasonography, Affiliated Union Hospital of Fujian Medical University, Fuzhou, China; 2 Department of Ultrasonography, Affiliated Sir Run Run Shaw Hospital of Zhejiang University, Hangzhou, China; 3 Department of Ultrasonography, People’s Hospital of Zhuhai City, Zhuhai, China; 4 Department of Pharmacy, Affiliated Union Hospital of Fujian Medical University, Fuzhou, China; The Ohio State University, United States of America

## Abstract

**Purpose:**

The distribution of targeted nanoparticles in tumor tissue is affected by a combination of various factors such as the physicochemical properties of the nanoparticles, tumor hemoperfusion and tumor vascular permeability. In this study, the impact of the biological effects of ultrasound on nanoparticle targeting to liver carcinoma was explored.

**Methods:**

The copolymer MePEG-PLGA was used to prepare the galactosylated docetaxel nanoparticles (GDN), and the physical and chemical properties as well as the acute toxicity were then assayed. The impact of ultrasound exposure (UE) on tumor hemoperfusion was observed by contrast-enhanced ultrasonography (CEUS), and the distribution of docetaxel in tumors and liver were detected by high performance liquid chromatography (HPLC). In the GDN combined with UE treatment group, the mice were injected intravenously with GDN, followed by ultrasound exposure on the human hepatocellular carcinoma xenografts. Twenty-eight days post-administration, the tumor growth inhibition rate was calculated, and the expression of Survivin and Ki67 in tumor tissues were determined by immunohistochemistry assay and quantitative real-time PCR.

**Results:**

The mean size of prepared liver-targeting nanoparticles GDN was 209.3 nm, and the encapsulation efficiency was 72.28%. The median lethal dose of GDN was detected as 219.5 mg/kg which was about four times higher than that of docetaxel. After ultrasound exposure, the tumor peak - base intensity difference value, examined by CEUS, increased significantly. The drug content in the tumor was 1.96 times higher than in the GDN treated control. *In vivo*, GDN intravenous injection combined with ultrasound exposure therapy achieved the best anti-tumor effect with a tumor growth inhibition rate of 74.2%, and the expression of Survivin and Ki67 were significantly decreased as well.

**Conclusion:**

Ultrasound exposure can improve targeting nanoparticles accumulation in the tumor, and achieve a synergism antitumor effect on the hepatocellular carcinoma xenografts.

## Introduction

Hepatocellular carcinoma is one of the most common malignant tumors in the world, with a global incidence rate exceeds 626,000 per year, ranking it third among the most lethal malignant tumors [1]. At present, the standard treatment for hepatocellular carcinoma includes surgical resection and minimally invasive treatment. However, due to multiple nidi and rapid deterioration from the disease, only about 30% of patients can take advantage of surgical resection or liver transplantation [2]. For most inoperable patients, transcatheter arterial chemoembolization is the preferred treatment, yet the 5-year survival rate is merely 26% [3]. In recent years, tumor targeting therapy has advanced due to its high efficacy and fewer side effects [4]–[6]. In theory, conjugating ligands or antibodies to nanoparticles can selectively transport the chemotherapeutics to the targeted tumor. Yet, the biodistribution of targeted nanoparticles after intravenous injection is influenced by various factors, such as reticuloendothelial system elimination, the physicochemical properties of the nanoparticles, the size of vascular endothelial pores, and the tumor hemoperfusion. Xu prepared galactosylated docetaxel nanoparticles and studied their distribution, finding that the amount of drug accumulating in hepatocellular carcinoma xenografts was much lower than in the liver, or even in the kidney [7]. Hence, improving the accumulation of targeted drug in the tumor is the goal of hepatocellular carcinoma targeted therapy.

**Figure 1:**
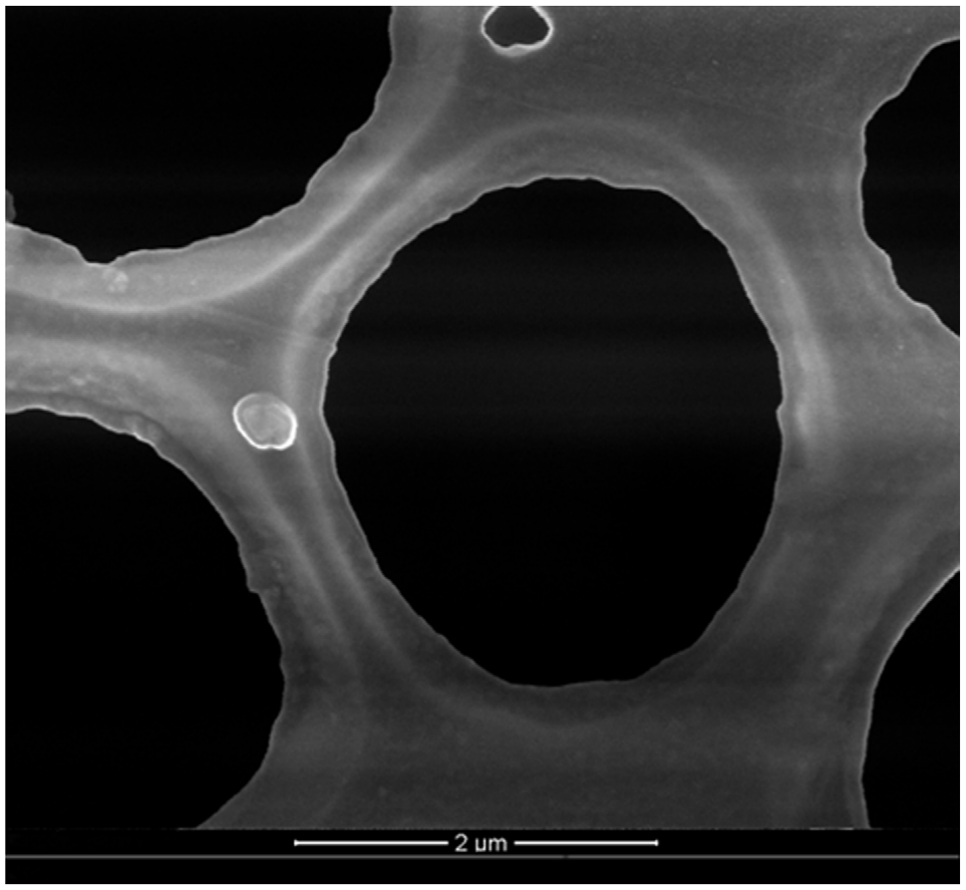
Scanning electron microscopy photograph of GDN (50 000×). GDN prepared by the method of modified emulsification-solvent evaporation were spheroid in shape with no coalescing, and the mean size was detected as 209.3 nm.

The transmission of ultrasonic waves in biological tissues produces thermal effects, acoustic cavitation, mechanical effects, and other modifications, which were found to help improve the anticancer effects [8]–[10]. With acoustic energy increases, the biological effects including the raised tissue temperature and improved vascular and cell permeability help the conventional drugs transport through the vascular and cell membrane. However, the drug transportation has bidirectional property; that is the drugs can either transport to the tumor cells or return to the blood circulation. In this study liver targeted nanoparticles were prepared and ultrasound exposure was used to help increase the vascular permeability and improve targeted nanoparticles transport to the liver cancer cells. Nanoparticles can then bind to and enter the cells by endocytosis, further improving the tumor targeting therapy effects.

## Materials and Methods

### Chemicals

Cholesterol, polyvinyl alcohol (Sinopharm Chemical Reagent Co., Ltd., China); Docetaxel crude drug (purity>99.5%, HONCH Pharmaceutical Co., Ltd., Hubei, China); polyethylene glycol monomethyl ether-poly (lactic acid-glycolic acid) copolymer (MePEG-PLGA) (Daigang Bioengineering Co., Ltd., Shandong, China), sonographic contrast agent SonoVue (Bracco SpA, Milan, Italy), Survivin and Ki67 polyclonal antibodies (Maixin-Bio, Fuzhou, China).

### Preparation and Characterization of GDN

The galactosylated cholesterol, cholesten-5-yloxy-N-(4-((1-imino-c-b-D- thiogalactosylethyl) amino) butyl) formamide (Gal-C4-Chol) was synthesized by the method described previously [11], [12], which targets to the asialoglysoprotein receptor on the hepatoma cell membrane. GDN were prepared using the method of modified emulsification-solvent evaporation. MePEG-PLGA 100 mg, docetaxel 5 mg and Gal-C4-Chol 15 mg were dissolved in 1 ml dichloromethane, then injected into 5 ml of 3% polyvinyl alcohol aqueous solution, and sonicated at 50W for 30sec, repeating five times. Then 10 ml of distilled water was added and the mixture magnetically stirred to remove the dichloromethane. The mixture was centrifugated at 15 000×g for 10 min, and then we collected the precipitation as GDN, that was vacuum freeze-dried and sterilized by Co^60^ irradiation. Docetaxel nanoparticles (DN) were prepared without Gal-C4-Chol.

The particle size of GDN was measured by a ZETASIZER3000HS laser particle size analyzer *(*Malvem Instruments Ltd, UK*)*. The superficial morphology was observed using scanning electron microscopy. The encapsulation efficiency was determined by HPLC at 30°C with a mobile phase of methanol/H_2_O (75/25, v/v) and a flow rate of 1.0 ml/min.

### *In vitro* Release of GDN

Five mg of GDN in 50 ml phosphate buffer solution (pH 7.2, including 0.02% sodium azide and 0.1% Tween 80) was placed in an orbital shaker and maintained at 37°C and 80 rpm for measurement of in vitro release. At 3, 6, 12, 24, 48 and 72 hrs, samples were centrifuged using a centrifugal filter device at 5000×g for 10 min, then the filtrates were collected to detect the docetaxel content by HPLC.

### Ethics Statement

This study was carried out in strict accordance with the Guidelines of the Animal Care and Use of Fujian Medical University. The protocol was approved by the Committee on the Ethics of Fujian Medical University. All efforts were made to minimize suffering.

### Acute Toxicity Test

Fifty male and 50 female Kunming mice weighing 18–22 g were randomly divided into two groups, the docetaxel and GDN groups. Each group was then divided into five dosage treatment groups of 10 mice. The drugs were injected via the tail vein followed by observations for eight weeks. Toxic reactions and the number of deaths were recorded, and the median lethal dose (LD_50_) was calculated according to the modified Karber’s method [13].

### Influence of Ultrasound Exposure on Tumor Temperature

BALB/c nu-nu male nude mice, weighing 16–18 g, were provided by Shanghai SLAC Laboratory Animal Center. The mice were kept in a pathogen free environment, and allowed free access to chow and water.

The human hepatoma cell line HepG2 (Shanghai Cell Bank, Chinese Academy of Sciences) was grown in RPMI-1640 medium containing 10% fetal bovine serum at 37°C under 5% CO_2_ and saturated humidity. Cells were collected and adjusted to a density of 1×10^7/^ml, and 0.2 ml injected into the right armpit of nude mice to create the model of hepatocellular carcinoma.

Twelve hepatoma-bearing nude mice with a tumor diameter of 1.7–2.0 cm were used to detect the influence of ultrasound exposure (frequency 840 kHz, acoustic intensity 0.75w/cm^2^) on tumor temperature. The ultrasonic probe covered the whole tumor, the exposure time was set at 1, 2, 3 and 4 min and the tumor temperature was detected using an electronic thermometer, and used to create the time-temperature curve.

### Tumor Contrast-enhanced Ultrasonography

Twelve hepatoma-bearing mice with a tumor diameter of 1.7–2.0 cm were divided into two groups, control group and UE group (n = 6 each). In the control group, acoustic contrast agent SonoVue (5 ml/kg), a sulfur hexafluoride-filled microbubble, was injected by bolus via the tail vein, and then the tumor was treated by CEUS (SEQUOIA 512, Siemens, center probe frequency 10 MHz, mechanical index 0.25). In the UE group, the tumors were subjected to ultrasound exposure for 2 min, and followed by CEUS examination. The contract-enhanced index, arriving time (AT), time to peak (TTP), and peak-base intensity difference (PBD) were determined by the quantitative analysis software, Syngo US Workplace.

### Biodistribution

Twenty hepatoma-bearing nude mice with a tumor diameter of 1.2–1.5 cm were randomly assigned to four groups (n = 5), DN, GDN, DN combined with UE and GDN combined with UE. In all groups, the mice were injected intravenously with a docetaxel dose of 10 mg/kg. The mice in groups combined with UE achieved additional ultrasound exposure for 2 min every two hours, repeating three times, right after DN or GDN injection. Two hours after the last treatment, the mice were sacrificed by exsanguination, and the tumor and liver were collected. The tissues were homogenized with 50% methyl cyanides, centrifuged at 12, 000×g for 10 minutes, and the supernatant sampled to determine the docetaxel content using an HPLC assay.

### *In vivo* Antitumor Effects

Twenty-five mice with a tumor diameter of 1.2–1.5 cm were randomly divided into five groups (n = 5). The mice were anaesthetized using ether and received treatment every week, the doses of DN and GDN were calculated according to the encapsulated docetaxel dose of 10 mg/kg.

1. model group.
2. DN group, in which the mice were intravenously injected with a DN suspension.
3. GDN group, intravenously injected GDN suspension.
4. DN+UE group, intravenously injected DN suspension, followed by UE for 2 min every two hours, repeating three times.
5. GDN+UE, intravenously injected GDN suspension, followed by UE.

Every week, the length (*L*), width (*W*) and thickness (*T*) of the tumors were measured by ultrasonography. Tumor volume was calculated according to the formula: V(mm^3^) = 1/2 (*L*×*W*×*T*). Data was plotted to analyze the tumor growth. At the end of the experiment, twenty-eight days post-administration, the mice were sacrificed and the tumors were removed and weighed to calculate the tumor growth inhibition rate according to the formula: IR (%) = [1-(mean tumor mass in the treatment group/mean tumor mass in model group)] × 100%.

### Immunohistochemistry Examination

The tumors were dissected to perform immunohistochemistry. The expression of Survivin and Ki67 in tumor cells were detected by the SP method. The primary antibodies, mouse anti-human polyclonal antibodies were used to detect the expression of Survivin and Ki67. Negative-control sections were incubated in blocking buffer alone without primary antibody. Each section underwent 3, 3′-diaminobenzidene staining, and haematoxylin re-staining. With a 200 times magnification, in 10 microscopic fields, the ratio of positive stained tumor cells to the total tumor cells was calculated for analyses.

### Expression of Survivin and Ki67 by Quantitative Real-time PCR

According to the manufacturer’s instructions, tumor total RNA was extracted and reverse transcribed to cDNA. The SmRNA expression of Survivin and Ki67 were determined by the real-time fluorescence quantitative PCR method on an ABI 7300 PCR instrument. Each reaction was run in triplicate and contained 2 µl of template, along with 1.6 µl primers in a final reaction volume of 25 µl. The sequences of the primer pair for Survivin were 5′- CAG ATT TGA ATC GCG GGA CCC -3′ (sense) and 5′- CCA AGT CTG GCT CGT TCT CAG -3′ (antisense) (expected size: 208 bp). The sequence of the primer pair for Ki67 were 5′-AAC ACC TAC AAA ATG ACT TCT-3′ (sense) and 5′-CTT CAC TCT TAC TTT CCA CAG-3′ (antisense) (expected size: 146 bp). The sequence of the primer pair for GAPDH were 5′- GCA CCG TCA AGG CTG AGA AC -3′ (sense) and 5′- ATG GTG GTG AAG ACG CCA GT-3′ (antisense) (expected size: 143 bp). Cycling parameters were 95°C for 2 min, following by 40 cycles of 95°C for 30s, 60°C for 20s, and 72°C for 20s. Data were obtained as average CT values and normalized against control as △CT. Expression changes in genes transcript between model and treatment group are shown as 2^△△^CT.

### Statistical Analysis

All data are presented as mean ± standard deviation using SPSS 16.0 statistical software package. A Mann-Whitney U test and single-factor analysis of variance were carried out, and *P*<0.05 is used to indicate a significant difference and *P*<0.01 a very significant difference.

## Results

### Characteristics of GDN

The prepared GDN were spheroid shaped (Figure1), with a mean size of 209.3 nm and polydispersivity index of 0.40. The drug encapsulation efficiency was detected as 72.28%.

### *In vitro* Release of GDN

**Figure 2:**
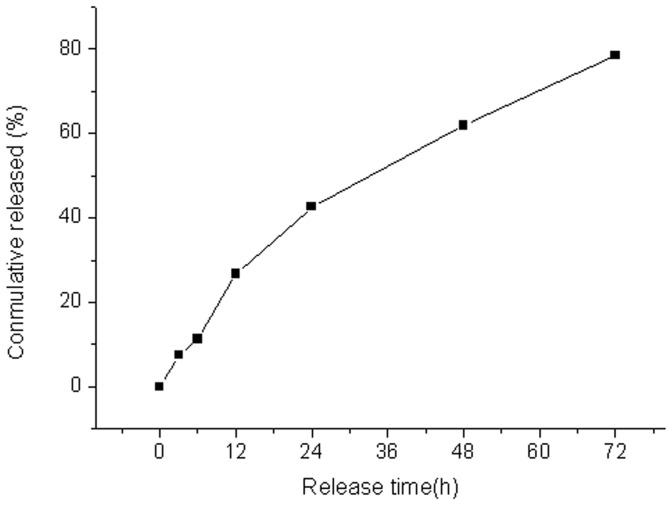
In vitro release curve of GDN. The prepared nanoparticles in vitro released the drug steadily for 72 hours without occurrence of burst release.

The in vitro release of GDN was steady and gradual with an accumulated release of 78.5% in 72 hours. There was no initial burst of drug release (Figure 2).

### Acute Toxicity Test

The LD_50_ of docetaxel was determined to be 47.3 mg/kg. The LD_50_ of the GDN was significantly higher, 219.5 mg/kg.

### Influence of Ultrasound Exposure on Tumor Temperature

**Figure 3:**
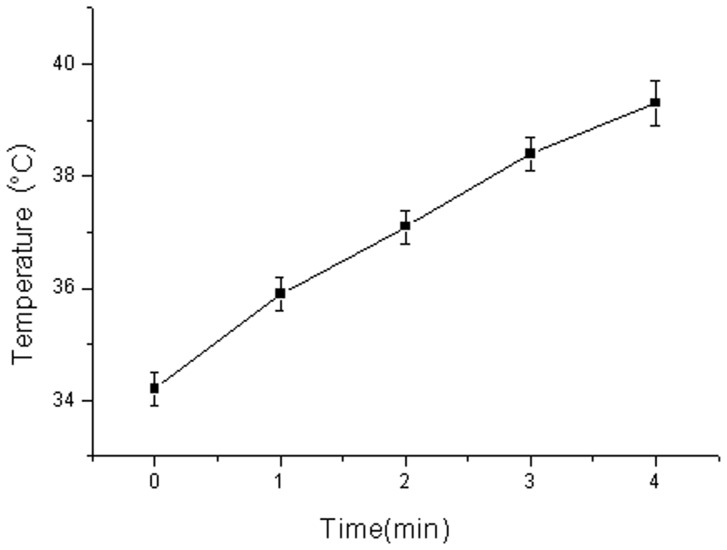
Influence of ultrasound exposure on tumor temperature. After ultrasound exposure, the tumor temperature increased steadily in a time dependent manner.

As showed in Figure 3, as the time of ultrasound exposure was extended, the tumor temperature increased steadily. After exposure for 4 min, the tumor temperature reached 39.3°C, which was 5.1°C higher than the pretreatment temperature.

### Biodistribution

**Figure 4:**
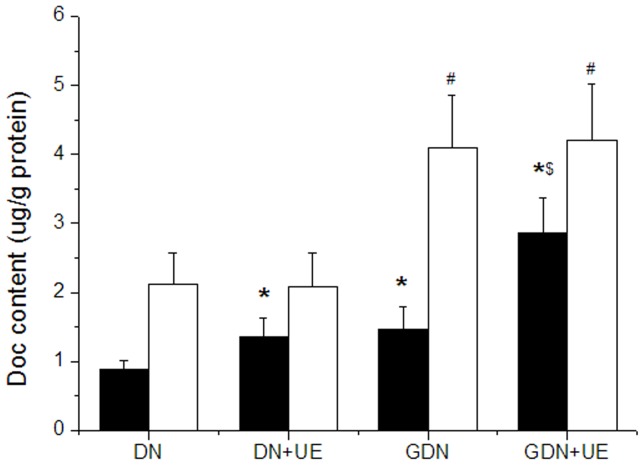
The biodistribution of docetaxel with different treatment was detected by HPLC. Mice were intravenously injected with the drugs, and in DN+UE and GDN+UE groups the tumors received additional ultrasonic exposure. Docetaxel content in the tumor of GDN, DN+UE and GDN+UE groups were significantly higher than in DN group (*P<0.05 *vs.* DN, ^$^ *P*<0.05 *vs.* GDN). In liver tissue, the docetaxel content in the GDN and GDN+UE groups were higher than that in DN group (^#^ *P*<0.05). ■ tumor, □ liver.

The content of docetaxel accumulating in the hepatoma xenograft in the GDN group was detected as 1.46±0.33 µg/g protein, which was higher than in the DN group 0.89±0.12 µg/g protein (P<0.05). After ultrasound exposure, the drug content in DN combined with UE increased to 1.35±0.29 µg/g protein, which was about 1.52 times higher than that in DN group (P<0.05). In GDN combined with UE group, the drug content increased significantly, reaching 2.86±0.52 µg/g protein, which was 1.96 times higher than that in GDN group (P<0.05) (Figure 4).

### Contrast-enhanced Ultrasonography

After intravenous injection of SonoVue, the tumors in both control and UE groups showed rapid and homogeneous enhancement, lasting about 90 sec. Compared to the control group, the values of AT and TTP in the UE group were slightly lower, while the PBD was much higher, and the difference was significant (*P*<0.05) (Table 1).

**Table 1:** Comparison of the tumor contrast-enhanced index (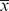
±s).

Group	AT (s)	TTP (s)	PBD (dB/cm^2^)
Control	1.89±0.60	4.21±1.32	21.61±6.83
UE	1.71±0.58	4.01±1.14	39.78±9.35*

* *P*<0.05 *vs*. Control group.

### *In vivo* Antitumor Effects

**Figure 5:**
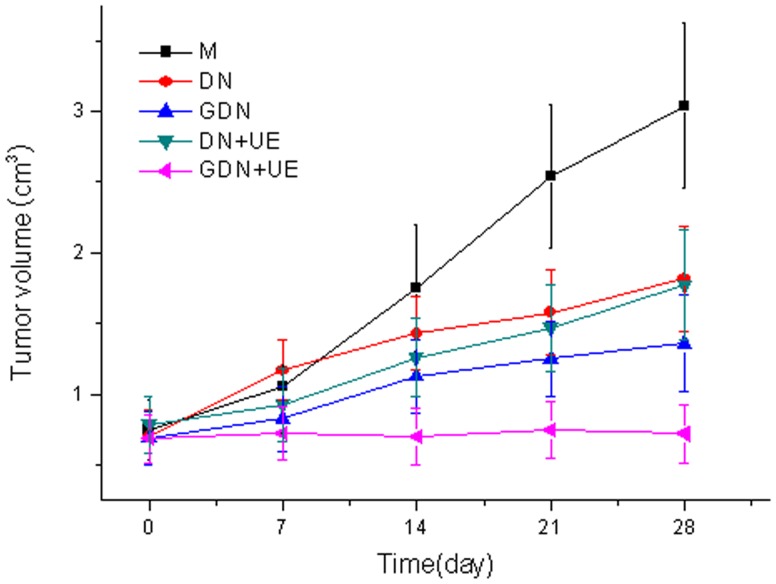
The growth curve of hepatocellular carcinoma xenografts. The tumor volume increased persistently in model group, while in all the docetaxel treated groups, the tumor volume increment was inhibited, especially in the GDN+UE group.

Hepatoma cell tumors grew continually following subcutaneous transplantation in nude mice. Compared to the model group, the tumor volumes were smaller in all the docetaxel treated groups, especially in the GDN+UE group (Figure 5).

Table 2 shows that all treatments inhibited tumor growth, in particular, the GDN+UE group displayed the most powerful antitumor effect, with a significant difference compared with all other groups (*P*<0.05 or *P<*0.01).

**Table 2:** Antitumor effect on hepatoma-bearing mice (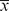
±s).

Group	Tumor mass (g)	IR (%)
M	3.21±0.43	–
DN	1.98±0.43**^##^	38.3
GDN	1.41±0.39**#	56.7
DN +UE	1.83±0.48* ^##^	43.0
GDN+UE	0.83±0.26**	74.2

* *P*<0.05 and **P<0.01 vs. M group.

# *P*<0.05 and ^##^ *P*<0.01 vs. GDN+UE group.

### Immunohistochemistry

**Figure 6:**
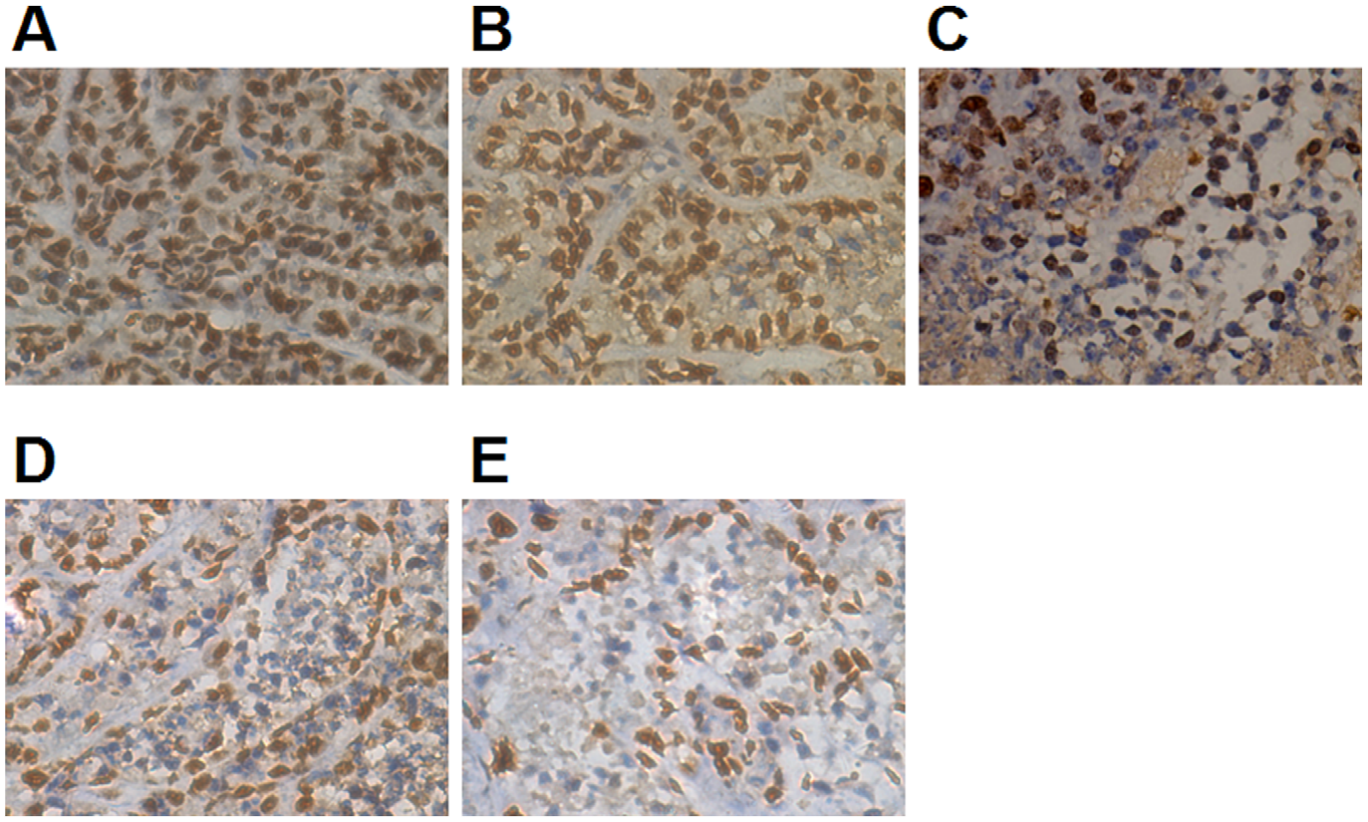
Expression of Survivin in tumors was detected by immunohistochemistry. After administration, the expression of Survivin was suppressed, especially in the GDN+UE group. A:model, B:DN, C: GDN, D:DN+UE, E:GDN+UE. See Table 3 for further explanation.

**Figure 7:**
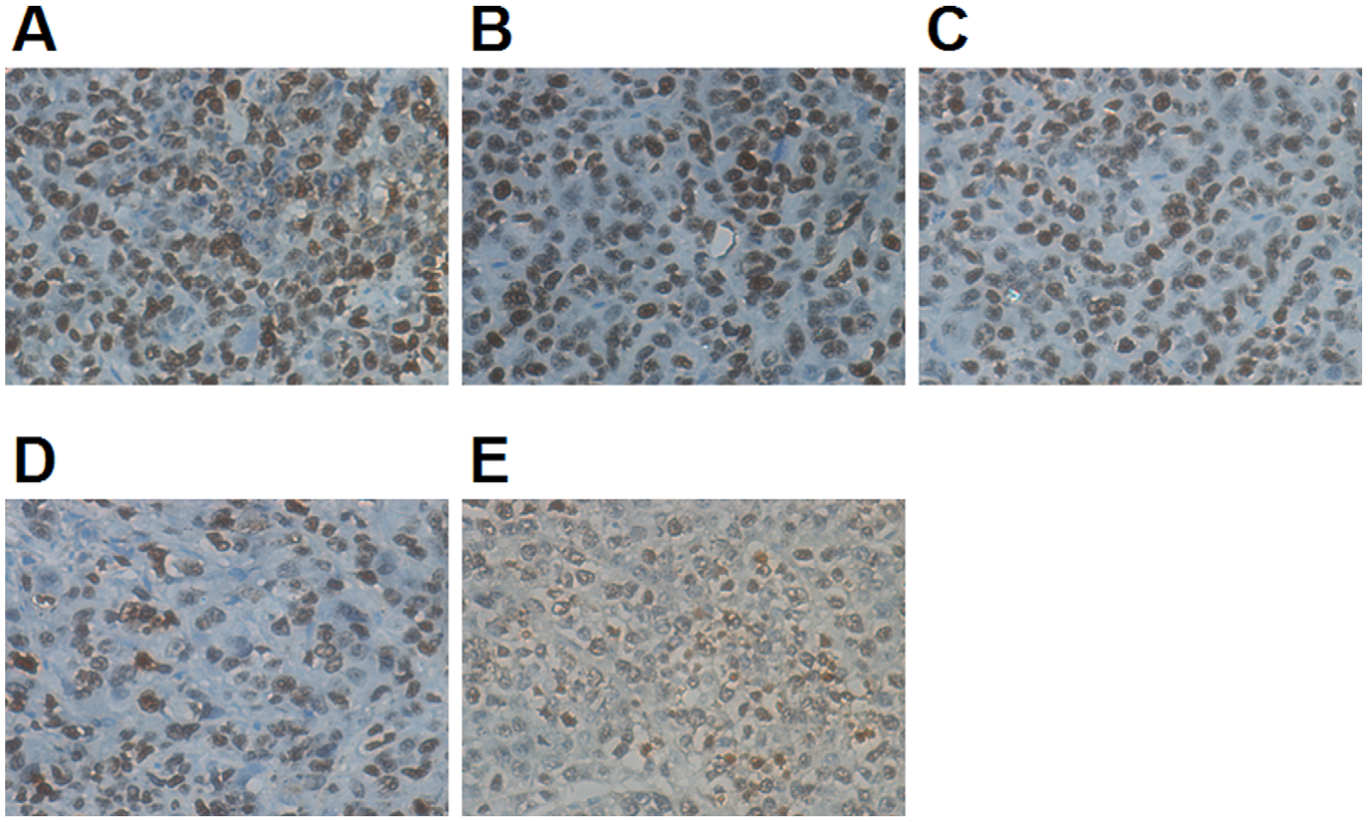
Expression of Ki67 in tumors was detected by immunohistochemistry. After treatment the expression decreased significantly, especially in the GDN+UE group. A:model, B:DN, C: GDN, D:DN+UE, E:GDN+UE. See Table 3 for further explanation.

In the GDN injection combined with ultrasound exposure treatment group, the expression of Survivin, an apoptosis inhibitor marker, and Ki67, a proliferation marker, in the tumor decreased. The percentage of positively stained tumor cells was lower than in the other groups, and the difference was significant compared with all other groups (*P*<0.05 or *P<*0.01) (Table 3, Figure 6 and 7).

**Table 3:** Expression of Survivin and Ki67 by immunohistochemistry (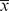
±s).

Group	Survivin (%)	Ki67 (%)
M	92.6±21.3	87.5±20.4
DN	69.2±19.6* ^##^	54.3±12.6* ^##^
GDN	51.7±16.4**#	44.9±9.5**#
DN+UE	45.9±10.5**#	43.5±6.9**#
GDN+UE	31.8±8.6**	28.7±5.7**

* *P*<0.05 and **P<0.01 vs. M group.

# *P*<0.05 and ^##^ *P*<0.01 vs. GDN+UE group.

### Determination of Survivin and Ki67 SmRNA Expression

The SmRNA expression of Survivin and Ki67 were significantly down-regulated in all docetaxel treated groups compared with control group, especially the GDN+UE treated group (*P*<0.01). Compared with the GDN+UE group, the difference was highly statistically significant for the DN group (*P*<0.01), and there was also a significant difference for the GDN and DN+UE groups (*P*<0.05) (Table 4).

**Table 4:** Expression of Survivin and Ki67 by real-time quantitative PCR (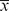
±s).

Group	Survivin	Ki67
M	1.00±0.33	1.00±0.36
DN	0.71±0.28* ^##^	0.67±0.29* ^##^
GDN	0.55±0.20**#	0.48±0.18**#
DN+UE	0.57±0.23**#	0.55±0.21**#
GDN+UE	0.37±0.16**	0.31±0.15**

* *P*<0.05 and **P<0.01 vs. M group.

# *P*<0.05 and ^##^ *P*<0.01 vs. GDN+UE group.

## Discussion

As an efficient chemotherapeutics with a wide antitumor spectrum, docetaxel can inhibit cell microtubule depolymerization, the synthesis of DNA, RNA or protein, leading to necrocytosis, and has been widely applied in treatment of breast cancer, ovarian cancer, non-small cell lung cancer, pancreatic cancer and others tumors [14]–[17]. However, docetaxel has a very poor water-soluble performance. Currently, Tween 80 is often used in clinical preparation to assist with water solubility, which tends to induce allergic reactions [18]. In this study, the preparation of targeted nanoparticles with the carrier of MePEG-PLGA resolved the water solubility problem and thus avoided allergic reactions induced by the solvent. The prepared targeting nanoparticles released the drug with the biodegradation of the MePEG-PLGA, and showed certain sustained release function, which may be the main contributor of significant reduction of the lethal dose 50 in mice.

Generally, nanoparticle with small size, good hydrophilicity and low zeta potential can effectively avoid identification and elimination by the reticuloendothelial system, and extend their plasma half-life [19]. In this study, the amphiphilic MePEG-PLGA was used to prepare the targeted nanoparticles. PEG is located in the outer shell and improves its hydrophilic performance; thereby effectively avoiding clearance by the reticuloendothelial system. The vascular pore in normal tissue is less than 100 nm, yet due to the incomplete vascular structure, the pore is increased to about 700 nm in malignant tissue [20]. The mean size of the galactosylated docetaxel nanoparticles was about 209.3 nm, which was the range between the normal tissue and the malignant tumor vascular pore. After intravenous injection, more nanoparticles can aggregate in malignant tissues and obtain a better passive drug targeting effect.

The asialoglysoprotein receptor (ASGPR) was initially discovered and characterized in mammalian liver by Ashwell [21]. The receptor specifically binds glycoproteins containing terminal galactosyl groups on their oligosaccharide chain [22]–[24]. Nanoparticles conjugated with a galactosyl group can positively target liver or hepatocellular carcinoma, and enter the cells by endocytosis mediated by ASGPR [25], [26]. The prepared galactosylated docetaxel nanoparticles displayed a good liver targeting effect, after intravenous injection, the content of GDN accumulating in hepatocellular carcinoma was 1.64 times higher than that of docetaxel nanoparticles without galactosylation.

*In vivo*, targeted nanoparticles should first permeate the vascular wall and aggregate in the tumor tissue, then bind to the receptors and enter the cells. The result of biodistribution of galactosylated docetaxel nanoparticles showed that the amount of drug accumulating in the liver was much higher than that in the hepatoma xenograft; the tissue hemoperfusion and vascular permeability may be the main reason.

A large number of studies have indicated that ultrasound exposure can increase tissue temperature in an acoustic intensity and time dependent manner. In this study, the acoustic intensity was set as 0.75W/cm^2^. After ultrasound exposure for 2 minutes, the tumor temperature was increased by 2.9°C. When the exposure time rose to 4 minutes, the temperature increase reached 5.1°C, which would injure the skin of the mouse. So an ideal ultrasound parameter combination to improve tumor targeting therapy effects would be an acoustic intensity of 0.75W/cm^2^ with an exposure time of 2 minutes.

After ultrasound exposure, the peak-base intensity difference value, examined by CEUS, increased significantly, indicating that ultrasound exposure can improve the tumor blood supply. And the drug content in tumor tissue increased remarkably, further demonstrating that ultrasound exposure can enhance the tumor hemoperfusion, increase the vascular permeability, and thus improve the targeted nanoparticles accumulation in the tumor.

Ultrasound exposure significantly improved the antitumor effect of galactosylated docetaxel nanoparticles on the hepatoma xenograft, which showed a tumor growth inhibition rate of 74.2%. It also inhibited the expressions of Survivin and Ki67 in hepatoma cells. Survivin is the strongest apoptosis inhibitor in the currently known family of apoptosis inhibitor protein, and is highly expressed in liver cancer tissues [27]–[29]. Meanwhile, Ki67 is a marker with the highest sensitivity in the appraisal of cell proliferation and can satisfactorily reflect the differentiation of liver cancer cells [30]–[32]. The over-expressed Survivin and Ki67 is closely related with the biological characteristics of hepatocellular carcinoma; such as peplos invasion, tumor metastasis, and patient prognosis.

In conclusion, ultrasound exposure can produce bioeffects, and raise the tumor hemoperfusion, increase vascular permeability and the drug content in tumor tissues, hence improve the antitumor effects of galactosylated docetaxel nanoparticles on hepatoma xenografts.
